# Ketamine—From an Anesthetic to a Psychiatric Drug: Mechanisms of Action, Clinical Applications and Potential Risks

**DOI:** 10.3390/molecules30132824

**Published:** 2025-06-30

**Authors:** Ewa Gibuła-Tarłowska, Anna Wiszniewska, Magdalena Turyk, Paulina Szymczyk, Jolanta H. Kotlińska, Ewa Kędzierska

**Affiliations:** 1 Department of Pharmacology and Pharmacodynamics, Faculty of Pharmacy, Medical University of Lublin, 20-093 Lublin, Poland; ewa.gibula-tarlowska@umlub.pl (E.G.-T.); jolanta.kotlinska@umlub.pl (J.H.K.); 2 Student Scientific Club “Synapse”, Department of Pharmacology and Pharmacodynamics, Faculty of Pharmacy, Medical University of Lublin, 20-093 Lublin, Poland; a.wiszniewska@onet.eu (A.W.); magda.turyk2001@onet.pl (M.T.); paulinaszymczyk7@op.pl (P.S.)

**Keywords:** ketamine, anesthesia, pain, addiction, depression

## Abstract

Ketamine, originally developed as an anesthetic, is gaining increasing attention due to its multifaceted pharmacological properties. In addition to its use in anesthesia, ketamine exerts potent analgesic effects via N-methyl-D-aspartate (NMDA) receptor antagonism, modulating pain perception and reducing central sensitization, particularly in chronic and neuropathic pain conditions. Emerging evidence also supports ketamine’s potential in the treatment of substance use disorder, where it may disrupt maladaptive reward-related memories and promote neuroplasticity which facilitates behavioral change. Moreover, in recent years, S-ketamine has shown rapid and potent antidepressant effects, especially in treatment-resistant depression (TRD), probably due to increased glutamatergic signaling, synaptic plasticity and the release of neurotrophic factors. This heterogeneous therapeutic profile positions ketamine as a unique agent at the interface of anesthesia, pain management, addiction medicine and psychiatry, warranting further exploration into its mechanism and long-term effectiveness.

## 1. Basic Information

The medical history of ketamine begins in the 1950s, when a new class of anesthetic drugs—cyclohexylamines—was developed. The first representative of this group to be discovered was phencyclidine (PCP). However, due to its long duration of action, phencyclidine caused severe postoperative delirium in humans, which motivated scientists to search for a short-acting derivative with a more favorable pharmacological profile. Then in 1962, ketamine (2-(2-chlorophenyl)-2-(methylamino)cyclohexanone) was obtained by Professor Calvin Lee Stevens. One of the numerous derivatives of arylcyclohexylamine, ketamine showed unique anesthetic properties, combined with analgesic and amnestic effects and a short duration of action, making it a promising anesthetic drug [1]. The researchers found that, in contrast to its precursor, it did not impact blood pressure and did not cause major respiratory depression. In addition, it showed a number of advantages, such as a low incidence of postoperative nausea or vomiting and a lower psychomimetic potential. As a result of its observed effects, in 1970, the Food and Drug Administration (FDA) approved ketamine as an anesthetic drug for use in children and adults [2].

The etymology of the compound name ‘ketamine’ refers to its chemical structure, in which a ketone and an amine fragment can be distinguished. It is an arylcycloalkylamine consisting of a chlorophenyl ring linked to a cyclohexanone ring. Furthermore, ketamine constitutes a chiral molecule and exists as two enantiomeric forms—S-ketamine and R-ketamine—which differ in their pharmacological properties, potency and duration of action due to their distinct spatial configurations [3]. S-ketamine shows approximately three-times more powerful analgesic and anesthetic effects [4,5] with a three-times greater affinity for N-methyl-D-aspartate (NMDA) receptors [6,7]. However, commercially, it is available in a racemic form containing equal parts of both enantiomers in the form of the hydrochloride salt for parenteral administration [1,3,8] and as a single S-ketamine used intranasally, as this is cheaper [3,4].

**Figure 1:**
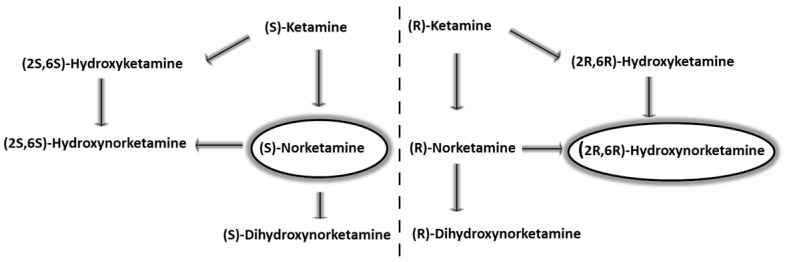
Metabolism of ketamine enantiomers.

Initially, ketamine was employed exclusively as an anesthetic. Its unique dissociative effects and partial agonism at mu opioid receptors allow for painful procedures to be performed in a continuous state of sedation and comfort [9,10,11]. The term ‘dissociative anesthetic’, which is used to describe ketamine, suggests that it induces a state of detachment from the environment in the patient, with an accompanying distortion of visual and auditory stimuli [1,12]. This state has often been described as floating in space and a lack of feeling in the arms and legs. Unfortunately, such effects have contributed to attempts at the recreational and criminal use of this drug [1]. Particularly, subanesthetic doses of ketamine can induce psychedelic effects which manifest as hallucinations and enhancements in sensory perception. Furthermore, it causes memory impairment, which increases according to the administered dose [13,14,15]. However, scientists have focused primarily on the potential therapeutic applications of ketamine. It has been noted that when administered at a subdissociative dose (0.1 to 0.4 mg/kg intravenously, i.v.), otherwise known as ‘low-dose ketamine’ (LDK), it has analgesic and sedative properties, so it can be effective in treating chronic, postoperative and neuropathic pain [3,12]. In this way, it is used either alone or as an adjunct to other pain relief medications. It is safe and effective in combination with injectable nonsteroidal pain medications, as well as opioids. Furthermore, as concerns about opioid use have grown, it has become more widely accepted [16,17]. The dosage determines the application and resulting effects of the drug, leading to variations in the prescribing protocol. This versatility renders ketamine valuable in both anesthesia and pain management. Currently, ketamine is an alternative hospital drug for the relief of postoperative pain and to prevent the exacerbation of acute or chronic pain when standard therapy fails. In addition, ketamine reduces opioid tolerance and prevents opioid-induced hypersensitivity (OIH), which can develop after the chronic use of opioids [12,15,18]. Since these conditions are mainly caused by the activation of NMDA receptors by opioids, they are reversed by ketamine administration [19]. Another extremely interesting finding was the antidepressant effect of ketamine. The rapid improvement observed in patient mood in the 1970s [1] now constitutes one of the main lines of research on the use of ketamine as an effective antidepressant [20]. Particularly important is the fact that the antidepressant action appears very quickly and persists for about a week even after a single administration. Both enantiomers of ketamine are rapidly metabolized in the liver by cytochrome P450 enzymes [21,22]. In the first step, S-ketamine and R-ketamine are demethylated by CYP3A4 or CYP2B6 to the main metabolites: S-norketamine and R-norketamine [23,24]. These compounds are then converted to dehydronorketamines (DHNKs) (S-DHNK and R-DHNK) or hydroxyketamines (HNKs) (S-HNK and R-HNK), respectively [25,26]. Additionally, cytochrome CYP2A6 catalyzes the hydroxylation of norketamines at position 6, forming (2S,6S)-HNK and (2R,6R)-HNK (Figure 1). Although the liver is considered the main site of ketamine metabolism, the kidneys, intestines and lungs are also potential metabolism sites [27]. The metabolites are excreted and eliminated in the urine and bile [28]. Furthermore, age-dependent differences in ketamine metabolism are observed. In pediatric cases, higher doses are required due to their accelerated metabolism. Conversely, in elderly individuals, a slower metabolic rate necessitates lower doses [29]. In view of the information presented above, it is essential to note the pharmacological differences in the activity of enantiomers and active metabolites of ketamine. While R-norketamine, an R-ketamine metabolite, does not show antidepressant activity, S-norketamine, the main metabolite of S-ketamine, induces a comparable antidepressant effect to the mother compound, with a slightly lower affinity for NMDA receptors. Furthermore, behavioral tests showed no potential for abuse or psychotomimetic effects compared to S-ketamine, making it a safer alternative in the treatment of depression. Nevertheless, further studies are needed to determine its potential in terms of this therapeutic indication [30,31].

## 2. Pharmacokinetic Properties

In addition to i.v., ketamine is commonly given via the intramuscular (i.m.) and subcutaneous (s.c.) routes. Ketamine demonstrates good solubility in both water and lipids; thus, it may be administered orally, rectally and even intranasally [2,3]. This is an important property, as in many clinical cases it may be impossible or dangerous to administer the drug by one of these available routes, necessitating the choice of another route. However, contradicting those advantages, routes of drug administration vary in bioavailability. Following i.v. administration, ketamine exhibits a rapid onset of action and peak plasma concentrations. Moreover, it rapidly enters the brain and then redistributes to peripheral tissues. The recommended initial i.v. dose of ketamine ranges from 1 to 4.5 mg/kg and should be administered over 60 s to individuals aged 16 and older. An average dose of 2 mg/kg typically induces analgesic or dissociative effects lasting about 5 to 15 min; the onset of action is within 10 to 30 s. When i.v. access is difficult, in emergency situations, i.m. or s.c. injections are used as an alternative. The i.m. route of administration, with an effective dosage of 8–10 mg/kg, provides a high bioavailability of 93% and results in peak plasma concentrations within 5 to 30 min [32,33]. However, oral ketamine has a much lower bioavailability of about 17% to 29% due to extensive first-pass hepatic metabolism, whereas the intranasal and rectal administration of ketamine demonstrate bioavailabilities of 45% to 50% and 25% to 30%, respectively [8] (Table 1). It is understandable that the selection of the appropriate route of administration of ketamine depends on many factors, including the patient’s condition, the type of procedure and the available resources. Through i.v., ketamine is employed for the induction of general anesthesia, acute pain control and diagnostic procedures, which guarantees a rapid onset of action, precise dosing and the possibility of continuous infusion. A separate method is the intranasal administration of S-ketamine in TRD, described later in the manuscript.

## 3. Molecular Mechanism of Action of Ketamine and Its Enantiomers (S-, R- and Racemic)

The mechanism of action of ketamine is multidirectional and not fully understood. Its primary mechanism involves non-selective and non-competitive antagonism of glutamatergic ionotropic NMDA receptors widely distributed in the central nervous system (CNS). NMDA receptors are heterotetrameric complexes typically composed of two GluN1 subunits combined with two regulatory GluN2 subunits (GluN2A-D), and sometimes GluN3 subunits. The specific composition of these subunits determines the receptor’s functional properties, including ion conductance, gating kinetics and sensitivity to pharmacological agents. NMDA receptors play a key role in synaptic transmission, plasticity and learning and memory processes [49,50]. Furthermore, ketamine differentially inhibits NMDA receptors depending on their subunit composition, with receptors containing GluN2C/D subunits displaying distinct sensitivity compared to those with GluN2A/B subunits [35,36,37,38,39,51]. At low concentrations, ketamine induces the allosteric inhibition of NMDA receptors. This allosteric action occurs through the stabilization of receptor conformations that reduce receptor activation independently of the channel-blocking effect, likely by binding to sites outside the ion channel pore. This mechanism, as described by [52], contributes to the fine-tuning of NMDA receptor activity and may underlie ketamine’s unique pharmacological profile, including its rapid anesthetic and antidepressant properties [52]. Ketamine also interacts with sigma receptors, particularly sigma-1 receptors that are chaperone proteins located in the endoplasmic reticulum membrane which are involved in neuroprotection, synaptic plasticity and cellular stress responses. Sigma-1 receptors modulate calcium signaling, ion channel activity and mitochondrial function. Ketamine acts as an agonist of these receptors, and this interaction may contribute to its antidepressant and neuroprotective effects, notably through the regulation of neurotrophic factors like brain-derived neurotrophic factor (BDNF) and the modulation of stress-related pathways [53,54]. Ketamine modulates adrenergic and serotoninergic receptors, influencing neurotransmitter release and mood regulation, thereby complementing its rapid antidepressant actions [55]. It also blocks voltage-gated calcium and potassium channels, further affecting neuronal excitability and synaptic signaling [40,41,56]. Acting as an H1 receptor antagonist, ketamine contributes to sedation and anesthesia [56].

Moreover, ketamine exhibits antihistaminic and anti-inflammatory (immunosuppressive) properties, as it can inhibit histamine release from mast cells, likely through the modulation of calcium-dependent processes. This antihistaminic action may play a role in reducing inflammatory responses during surgical procedures and chronic pain treatments [57]. Ketamine demonstrates the aforementioned significant anti-inflammatory effects by suppressing pro-inflammatory cytokine production, including interleukin-6 (IL-6), tumor necrosis factor alpha (TNFα) and Interleukin-1β (IL-1β). In addition, it can modulate key inflammatory signaling pathways, such as the inhibition of nuclear kappa B (NF-κB) activation, thereby downregulating genes involved in inflammatory responses. These properties are particularly relevant in conditions like chronic pain and depression, where inflammation plays a central role [30,58]. Furthermore, ketamine reduces the activation of macrophages and microglia, which may explain its neuroprotective properties in conditions associated with neuroinflammation [58,59].

Other pharmacological targets of ketamine include the inhibition of nicotinic acetylcholine receptors (nAChRs), particularly α7-nAChRs, which are involved in inflammatory and cognitive processes. This action may contribute to ketamine’s effects on memory and its anti-inflammatory profile [60]. Ketamine also interacts with opioid receptors, primarily as a weak partial agonist at µ-opioid receptors, which may enhance its analgesic properties, although the clinical relevance of this interaction remains debated [61]. These diverse actions highlight ketamine’s complex pharmacological profile, extending beyond NMDA receptor antagonism to include the modulation of sigma receptors, immune and histaminergic systems and additional molecular targets. This multifaceted activity likely underpins its wide-ranging therapeutic effects in clinical practice.

However, in this case, we observe high activity not only of the racemic form but also of the single enantiomers. S-ketamine has an approximately two-to-three-times greater affinity for NMDA receptors than R-ketamine, making it more potent as an anesthetic and analgesic [6]. Its higher potency allows for lower dosing, potentially reducing side effects while maintaining therapeutic efficacy. S-ketamine has also demonstrated a rapid antidepressant effect and is approved for use in TRD under the name ‘esketamine’ [62]. In addition to its primary effects on NMDA receptors, S-ketamine has been shown to affect α-amino-3-hydroxy-5-methyl-4-isoxazolepropionic acid (AMPA) receptor activity, contributing to synaptic plasticity and neuroprotection. This mechanism is thought to underlie its rapid antidepressant properties [46].

Although R-ketamine is less potent at NMDA receptors compared to its S-counterpart, it exhibits unique pharmacodynamic properties, with preclinical studies suggesting that R-ketamine may provide longer-lasting antidepressant effects due to stronger modulation of synaptic plasticity and neurogenesis [63]. Similarly, S-ketamine enhances synaptic plasticity and neurogenesis by increasing BDNF release and activating the mTOR signaling pathway, which supports synaptic remodeling and new neuronal formation, contributing to its rapid therapeutic effects [62,64].

The racemic mixture of ketamine contains both the S- and R-enantiomers, combining their respective pharmacological properties. This formulation is widely used in clinical practice for analgesia and pain management, as it provides a balanced profile of rapid analgesic, sedative and antidepressant effects [8]. The complementary actions of both enantiomers may also contribute to ketamine’s efficacy in conditions such as neuropathic pain and chronic pain syndromes. Clinically, the racemic form offers the advantage of utilizing the high affinity of S-ketamine for the NMDA receptor to achieve rapid effects, while incorporating the potentially longer-lasting therapeutic actions of R-ketamine. This duality may enhance the overall efficacy of ketamine, particularly in complex or refractory cases [62].

## 4. Clinical Uses in Anesthesiology

Focusing on the use of ketamine as an anesthetic, it should be noted that it is well entrenched in the induction and maintenance of general anesthesia, both inside and outside the operating room, as well as in emergency states. Ketamine is highly effective for short medical procedures that do not require skeletal muscle relaxation and can be used as a pre-anesthetic for the induction of general anesthesia in combination with other drugs, such as nitrous oxide in patients of different ages, ranging from 3 months of age [29] onwards. Of particular importance is the fact that it maintains proper skeletal muscle tone, preserves systemic vascular resistance and cardiac efficiency and does not affect oxygen saturation: it maintains proper pharyngeal and laryngeal reflexes and enables spontaneous breathing [65]. Moreover, ketamine maintains the reflexes of the respiratory system and exhibits bronchodilatatory effects—which makes it safe for patients with active bronchospasm and reactive airway disease [33,66], as well as asthma [67]. The drug is effective in states where hemodynamic balance is required—in trauma, sepsis or cyanotic heart disease. Ketamine is likewise valuable in total i.v. anesthesia for patients with cardiogenic shock, hypovolemia and pericardial tamponade [33,49]. Furthermore, it may be used as an LDK alongside other drugs, such as opiates, benzodiazepines or propofol, which allows a reduction in the dosage of administered drugs by up to 50%, which equals a lower risk of side effects [66]. However, it should be emphasized that the onset and duration of the drug’s effect varies depending on the route of administration. Moreover, the differences in the activity of the individual stereoisomeric forms are significant. S-ketamine has an approximately two-to-three-times greater affinity for NMDA receptors than R-ketamine and it has been confirmed to have a greater anesthetic potency and faster clearance, which results in a shorter recovery time [6]. In the randomized, double-blind study of Geisslinger et al. [68], when a racemic mixture was given, a statistically significantly lower clearance and volume of distribution was observed for R-ketamine compared with S-ketamine; therefore, the body eliminates R-ketamine more slowly and it likely remains more in the circulation than in the tissues, whereas S-ketamine’s pharmacokinetics are more predictable and stable, regardless of whether it is administered alone or as part of a racemic mixture; it has a faster onset of action and is also eliminated faster, allowing the patient to regain psychomotor skills more quickly [68,69].

Racemic ketamine is known worldwide and has been used in medicine since the 1960s. It has been clinically tested; its properties, dosage and profile of action have been determined; and it is also inexpensive. The better parameters of S-ketamine, i.e., greater potency and a better profile of side effects, are of little importance in anesthetic practice, because many groups of drugs are utilized in such procedures, and these mutually intensify their effects in the mechanism of hyperadditive synergism, and the side effects are less significant when the patient remains unconscious [5,8,70,71]. S-ketamine is relatively new, is patented in some countries (e.g., Germany, Austria) and is significantly more expensive, which is why racemic ketamine is still the drug of choice in situations where there is an urgent need for anesthesia [72].

## 5. Risks and Benefits

In view of the considerable amount of controversy surrounding ketamine, the risk–benefit factors need to be evaluated before application as an anesthetic (Table 2). Generally, it has been observed that ketamine possesses neurotoxic potential connected to neuronal cell apoptosis and necrosis [73]. However, the research results are contradictory, with one stating ketamine’s toxicity has not been demonstrated in humans—only in select animal models [73]—and the other reporting the negative effect as ‘dose-dependent’ [74]. As mentioned previously, the anesthetic’s bronchodilatatory property makes it a well-established drug for patients with airway diseases, such as asthma and bronchospasms. Nonetheless, the administration of ketamine should be monitored, as the medicine may carry the risk of respiratory hazards and blockage. In addition, it can cause cardiovascular perturbations and psychomimetic effects; hence, blood pressure and electrocardiogram measurements should also be taken during operations [73,74]. It is crucial to mention the limitations of ketamine use in light of its dissociative as well as pro-convulsant properties. Administration of the drug may be dangerous to patients with epilepsy (can cause seizures) and schizophrenia (may induce or worsen psychosis). In addition, during recovery, the patient may be characterized by excessive agitation caused by hallucinations after ketamine anesthesia. Furthermore, the use of this drug can be particularly problematic during certain treatments and operations, as the patient anesthetized with ketamine can make spontaneous and involuntary movements [32]. The anesthetic can also induce hallucinations, delirium and vivid dreams. Likewise, it distorts sensory input to the upper centers of the CNS, affecting pain and emotional responses, as well as memory [66,75]. It also increases cerebral blood flow, which has been a factor in ruling out the administration of the anesthetic in patients with cranial injuries. However, studies show that this effect can be negated by controlling ventilation and sedation during anesthesia, and by administering midazolam along with ketamine. This pairing has even been shown to be more effective than the combination of fentanyl and midazolam [32].

As a conclusion, it should be emphasized that despite its good reputation and popularity over the years, today, ketamine is no longer the preferred primary drug for anesthesia. However, its wide spectrum of advantages still makes it a valuable co-anesthetic. In modern medicine, the use of ketamine should be preceded by an assessment of benefits and risks on an individual basis for the patient [74]. Very valuable sources of information in this context are public assessment reports. These reports, published by the European Medicines Agency (EMA), provide patients, healthcare providers and public health institutions with comprehensive insight into data regarding the effectiveness and safety of drugs. Currently, EMA publishes European Public Assessment Reports (EPAR), including the report for Spravato^®^ ((S)-ketamine nasal spray) which was approved by the European Commission in 2019 (European Medicines Agency. Spravato (S-ketamine): European Public Assessment Report, 2019. Available from https://www.ema.europa.eu/en/medicines/human/EPAR/spravato, accessed on 26 May 2025). Other authorized medicinal products, such as Ketalar^®^ or Ketanest^®^, contain racemic ketamine and do not have EPAR. This indicates that they were not assessed through the centralized EMA procedure but were authorized either via national procedures or through the Mutual Recognition Procedure (MRP) in individual European Union member countries. In such cases, information on these medicines can be found through national regulatory authorities or pharmaceutical databases.

## 6. Ketamine in Pain Management

Ketamine plays a crucial role in pain management primarily by modulating NMDA receptor activity, thereby reducing central sensitization, interrupting the wind-up phenomenon and diminishing the formation of pain memory. At the spinal cord level, NMDA receptor activation contributes to the development of central sensitization and the wind-up phenomenon leading to an increased transmission of pain signals to higher brain centers [76]. NMDA receptors are also involved in the formation of pain memories through mechanisms of long-term potentiation (LTP). Activation of these receptors in brain regions such as the anterior cingulate cortex leads to lasting changes in synaptic transmission, which can result in chronic pain sensations, even after the original stimulus has subsided [60]. Ketamine acts by reducing central sensitization, the wind-up phenomenon and pain memory. Central sensitization occurs when the nervous system becomes hypersensitive to pain due to prolonged stimulation. This causes an exaggerated pain response, even to stimuli that normally do not cause pain [76,77]. Ketamine helps to prevent this by blocking NMDA receptors, which are key mediators of central sensitization. In this way, it reduces hyperexcitability in pain pathways, helping to control acute and chronic pain. The wind-up phenomenon is a process in which the repeated stimulation of pain pathways leads to an increased pain response over time. It occurs due to the continuous activation of NMDA receptors in the spinal cord, which amplifies pain signals and may contribute to the presence of acute pain, its progression to chronic pain, increased pain sensitivity (hyperalgesia) and even allodynia, when stimuli that are normally painless, such as touch, can be perceived as painful [78,79]. Studies have shown that ketamine has mild-to-moderate analgesic effects for a variety of chronic pain conditions (e.g., neuropathic pain, central pain syndromes, headaches and temporomandibular joint disorders) [63,79] (Table 3). Pain memory (the persistence of pain perception even after the initial injury has healed) is a common feature of chronic pain. Ketamine’s ability to modulate neuronal plasticity in areas of the brain and spinal cord responsible for pain processing plays a significant role in ‘resetting’ pain pathways, thereby reducing the persistence of chronic pain [80].

Ketamine also interacts with opioid receptors, particularly the μ-opioid receptor. Studies have shown that ketamine can potentiate μ-opioid receptor-dependent signaling, enhancing the analgesic effects of opioids, which may explain the ability of ketamine to improve the efficacy of opioids and reduce postoperative opioid requirements. Perioperative subanesthetic doses of ketamine were effective in postoperative pain control, reducing opioid requirements, while displaying minimal adverse effects [81,82]. This was seen in breast, obstetric, bariatric and orthopedic surgeries, while minimal effect was evident in pediatric and thoracic surgeries [83]. Another advantage of LDK is that it can reverse opioid tolerance, allowing for more effective pain relief [67,68,69,70] and reduce or oppose the clinical features of opioid-induced hyperalgesia (OIH), in which opioids paradoxically increase pain in patients receiving long-term opioid therapy [82,84,85]. The occurrence of OIH is associated with changes in the conformation of opioid receptors [86]. Several mechanisms of the development of this phenomenon are considered, with the glutamatergic mechanism associated with the activation of NMDA receptors being extremely important. NMDA receptor stimulation can occur under the influence of opioids [69,70,71,72] which, by binding to μ receptors, activate protein kinase C (PKC), which causes phosphorylation of the NMDA receptor. The NMDA receptor, at rest, is blocked by magnesium ions and its phosphorylation causes the attenuation of the magnesium blockade and an increase in the influence of calcium ions [87]. This phenomenon causes an increase in pain conduction [86].

**Table 3:** The selected examples of the use of ketamine in different types of pain.

Type of Pain	Administration Regimen/Key Features	Literature
Acute	**Post-traumatic pain in prehospital settings** fentanyl combined with low-dose ketamine (0.25–0.3 mg/kg) was administered, resulting in more effective and safer analgesia.	[88,89,90]
	**Postoperative pain in orthopedic surgery** ketamine was administered as an i.v. bolus at a dose of 0.5 mg/kg, followed by a continuous i.v. infusion at 0.25 mg/kg/h, which resulted in a reduction in hyperalgesia areas without side effects.	[90,91,92]
	**Pain in renal colic** ketamine was administered intranasally at a dose of 1 mg/kg, which resulted in a reduction in pain intensity.	[91,92,93]
	**Pain after third molar extraction** a single intranasal dose of ketamine (50 mg) provided significant pain relief lasting for 3 h post-administration.	[43,88,94]
	**Pain in acute traumatic conditions in children** ketamine was administered intranasally 0.7 mg/kg, with the option of an additional 0.3 mg/kg bolus if pain exceeded 50 mm on the VAS scale; this regimen resulted in effective pain control with minimal and transient adverse effects.	[95,96,97]
Chronic and neuropathic	**Chronic pain—cancer-related (opioid-resistant)** i.v. infusion of ketamine; dosage 0.25–0.6 mg/kg for 4–6 h daily for several days; reduction in pain in opioid-resistant patients and improvement in quality of life.	[98,99,100]
	**Chronic pain—CRPS (complex regional pain syndrome)** daily infusions of ketamine 0.35 mg/kg i.v. for 4 h a day for 10 days results in long-lasting pain relief (lasting up to 12 weeks) and improved limb function.	[54,94,101]
	**Neuropathic pain—chronic (e.g., diabetes, neuralgia)** i.v. infusion of ketamine 0.1–0.5 mg/kg/h for 4–6 h; reduction in pain intensity, improved quality of life, possibility of reducing opioid dosage.	[102,103,104]
	**Neuropathic pain—postherpetic neuralgia** i.v. infusion of 0.1–0.5 mg/kg/h for 4–6 h; reduction in pain and improvement in patient functioning.	[97,103,104]
Perioperative	**Orthopedic surgery—arthroplasties** i.v. bolus 0.5 mg/kg during induction of anesthesia, followed by continuous infusion at 0.25 mg/kg/h, which resulted in a reduction in pain management and reduced opioid therapy.	[69,105,106]
	**Spinal surgery** i.v. bolus dose of 0.15–0.25 mg/kg prior to anesthesia induction, resulting in reduced pain.	[107,108,109]
	**Laparoscopic procedures** Low-dose ketamine—0.25 mg/kg, i.v. as a single bolus or by continuous infusion, resulting in improved pain control.	[110,111,112]
	**Orthopedic limb surgery** Subanalgesic doses of ketamine—i.v. bolus 0.3 mg/kg followed by i.v. infusion 0.2 mg/kg/h, which provides a strong analgesic effect.	[113,114,115]
Cancer	**Breakthrough cancer pain** for sudden, severe pain, an additional dose of ketamine can be given sublingually or intranasally 10–50 mg; rapid pain relief, especially for patients who do not tolerate opioids well.	[116,117,118]
	**Advanced cancer pain—palliative therapy** ketamine can be used as an add-on to opioids to help control pain: i.v., either as a continuous infusion or as a bolus (single dose): continuous infusion 0.1–0.3 mg/kg per hour or bolus 0.25 mg/kg every 8 h; pain relief and improved quality of life, especially in patients with opioid resistance.	[119,120,121]

In 2020, ketamine was approved by the World Health Organization (WHO) as a drug for the treatment of refractory neuropathic pain, the use of which is intended to reduce the doses of opioids used and serve as an alternative for people with an increased tolerance to opioids. However, it should be remembered that ketamine is not a first-line agent; it is a strong drug, intended for people who do not benefit from other therapeutic methods.

## 7. Ketamine as Potential Therapy for Addiction

Despite long-term pharmacological research on the creation of new substances, addiction still remains an unresolved important social and clinical problem. Furthermore, due to the complex nature of this disease, currently available drugs possess only limited effectiveness. This therapy is significantly complicated by the common co-occurrence of depression with addictions [122,123,124,125,126,127]. It is well known that untreated depression may cause or intensify addiction, and taking psychoactive substances may intensify or even lead to the development of depression. Furthermore, high levels of depression and anxiety may predispose not only to addiction, but also to relapse even after a long period of abstinence [126,128,129,130]. Therefore, scientists and clinicians pay attention to the possible benefits of using antidepressants in the treatment of addictions, as drugs enhancing abstinence. It seems that ketamine could be an interesting alternative in the treatment of addictions, especially since in many cases, conventional antidepressants do not reduce alcohol consumption [131,132]. This is mainly explained by the fact that the effects of these drugs are delayed in time.

As confirmation of these assumptions, recent decades have brought many new reports about the use of ketamine beyond its previous indications. Increasing number of studies suggest that it has a multifaceted effect that can be used, among other purposes, in the treatment or at least alleviation of some symptoms of addiction. Moreover, ketamine is characterized by properties different from most typical antidepressants and has a unique mechanism of action, which suggests that it may improve the ability to establish and maintain abstinence in substance use disorders.

It was well known that diminished glutamatergic synaptic transmission and reduced plasticity are associated with addiction [133,134]. Therefore, ketamine, which acts primarily by influencing glutamatergic transmission, may be particularly helpful in this therapy. Thus far, it has been suggested that the possible mechanisms by which ketamine may have a beneficial effect in addiction include the following: (1) enhancement of neuroplasticity and neurogenesis, (2) disruption of relevant functional neural networks, (3) treating depressive symptoms, (4) blocking re-consolidation of drug-related memories, (5) provoking mystical experiences, (6) enhancing psychological therapy efficacy.

Existing models suggest that ketamine’s blockade of NMDA receptors increases synaptogenesis by stimulating protein synthesis and the insertion of AMPA receptors [83,133,135]. Hence, ketamine’s effects help to reverse the glutamatergic changes associated with depression and addiction. The fact that the administration of rapamycin (an mTOR antagonist) blocks both the ketamine-induced reduction in alcohol intake [135] and the antidepressant effects [84,136] suggests that the underlying mechanism of both effects might be the same: synaptogenesis. Moreover, a previous study showed that ketamine’s metabolites, (R,S)-norketamine and (2S,6S)-hydroxynorketamine, contribute to ketamine-mediated increase in mTOR signaling both in vivo in rats and in vitro [137]. Therefore, this represents a mechanism by which ketamine could redress an imbalance in addiction. However, there are also contradictory results published by Zanos et al. [125], which indicate that antidepressant effects are independent of mTOR levels. Therefore, further research is needed to assess the relationship between changes in synaptogenesis and any effects of ketamine administration.

In addition, it has been shown that addictions are connected to disorders of neurogenesis, especially in cortical and hippocampal structures which may influence levels of self-administration and the vulnerability to relapse [138,139]. This process may be related, among others, to a reduction in BDNF serum levels [140] observed, among others, in groups of people addicted to cocaine and heroin [141,142,143]. Ketamine has been previously shown to affect the level of BDNF, and this mechanism is one of the key factors in its antidepressant action [39,91,92,144]. Hence, its use may also be helpful in combating addictions through this mechanism [145,146].

Ketamine also disrupts drug-cue memories via the manipulation of reconsolidation processes [93,94,95,96,97,143,147]. Recent reviews have suggested that ketamine may be able to disrupt maladaptive appetitive memories [93,94,95]. It is interesting that many authors indicate the specific involvement of mystical experience as induced by ketamine in the therapeutic mechanism [93,94,95,96].

This suggests that the combination of ketamine with motivational enhancement therapy may be an effective pharmacotherapy for initiating and sustaining abstinence. Indeed, this type of therapy, known as ‘Ketamine Psychedelic Therapy’ (KPT), has been used, among others, in patients with drug addiction, but also in people with other types of addictions (e.g., food addiction) and mental disorders [148,149]. It was suggested that ketamine can provide a unique mental state that facilitates and enriches therapeutic experiences, which in turn may improve efficacy and lengthen treatment effects [35,95]. The authors suggest that the subjective psychedelic experience seemed to help the addict to undergo a cathartic process, improve relationships with the world and other people, maintain positive psychological changes and enhance self-awareness and personal growth [149,150,151,152,153]. These changes are considered as favorable for promoting abstinence. Furthermore, patients feel less depressed and anxious, more self-confident and more emotionally open. It should be emphasized that ketamine infusions facilitate psychological therapy presumably due an increase in synaptogenesis and neurogenesis, and thus improved learning of relapse-reducing strategies [154]. However, some researchers suggest that despite the very promising effects observed during the use of KPT, it should be pointed out that a ketamine-induced psychedelic experience may have only marginal and transitory beneficial effects in and of itself, no beneficial effects at all or may be harmful when ketamine is used in uncontrolled settings recreationally, hence leading to significant medical problems and addiction [155,156,157]. These findings suggest a new usefulness for ketamine in facilitating addiction treatment and reducing the risk of relapse, namely, by maintaining motivation for sobriety even in the face of stressors and challenges.

Collectively, these studies reveal that ketamine may improve the ability to establish and maintain abstinence in substance use disorder. The presented findings (Table 4) may have a significant impact on the development of new treatments of addiction, as addiction is a complex condition that currently presents challenges for successful treatment.

In addition to the indicated action in reducing the effects of addiction, ketamine is characterized by having a much more favorable dosing method compared to classic pharmacotherapy. Daily administration is not needed, and this significantly improves treatment, since it is less stigmatizing than the requirement to take daily medication. The primary problem may be the route of its administration. Most published data concern administration via injection, which significantly limits its use and increases the costs of treatment. The search for a less invasive method of administration that would also limit the need to use highly specialized centers and would therefore be available to a wider group of patients is still ongoing. Currently, it seems that intranasal administration will be the optimal method of administration and widely expand the availability of ketamine treatment [173]. Furthermore, in the case of attempts to introduce ketamine into the pharmacotherapy of addictions, evaluations of the optimal dose and frequency schedules are also needed. Most of the studies to date have used prior depression trial dosages of 0.5–0.8 mg/kg i.v. ketamine, although a few studies utilized doses of 2–2.5 mg/kg i.m.

However, despite its many advantages, the effects of ketamine require further research for the following reasons:

1. Some of the presented studies used only small populations of naïve individuals, lacked inactive placebo groups or were relatively homogeneous in terms of ethnicity, age and gender. Thus, the effects of ketamine administration early in life have not yet been clearly established, and it is possible that ketamine administration to adolescents for the treatment of depression may lead to an increased risk of addiction later in life.
2. In the case of ethanol addiction, a very important element is the occurrence of potential interactions and co-dependency especially in that ketamine has become popular as a recreational drug, sometimes used with alcoholic beverages or stimulants.
3. The issue of the addictive effect of ketamine and the possibility of its therapeutic use in controlled conditions without causing addiction also remains unresolved.

However, as previously stated, extensive clinical trials are essential for determining the optimal dosing strategies, identifying biomarkers linked to therapeutic outcomes and evaluating the long-term risks associated with repeated administration. Despite these challenges, the available data represent a promising advancement in understanding the potential role of ketamine in addiction treatment.

## 8. Present Primary Pharmacotherapy of Depression

Depressive disorders represent one of the greatest challenges of modern medicine and pharmacy. According to data released in 2021, depression affects approximately 280 million people worldwide and is one of the most common causes of life-limiting disability [174]. However, its pharmacotherapy is still problematic due to the frequent unresponsiveness of patients to the available treatments [175]. Treatment includes mainly drugs belonging to the selective serotonin reuptake inhibitor (SSRI) and serotonin norepinephrine reuptake inhibitor (SNRI) groups, with the mechanism of actions of these corresponding to monoaminergic theory. However, the disadvantages held by these drugs may affect the efficacy of pharmacotherapy, leading to discontinuation, as well as impairing some areas of functioning. Such drugs are characterized by latency of action, the potential for worsening of the patient’s condition at the start of pharmacotherapy and sexual or cognitive dysfunction. Furthermore, drugs from the SSRI group may prolong bleeding time and induce teratogenic effects. Despite their relative efficacy, they are met with treatment non-response in some patients [174]. It is estimated that more than 30% of all patients with major depressive disorder (MDD) do not achieve satisfactory treatment results, and this outcome is associated with a reduced quality of life and increased deaths from suicide. Furthermore, it is noted that depression resistance to at least two antidepressants indicated by standard pharmacotherapy—TRD—is an increasingly occurring problem and a serious challenge for pharmacotherapy [176]. The exact causes determining treatment resistance are still not known, but, presumably, inflammation within the CNS and impaired neuroplasticity are involved in this process [40,177]. The lack of efficacy of conventional therapy leads to a search for drugs with new therapeutic targets. Currently, drugs targeting the glutamatergic and γ-aminobutyric acid (GABA)-ergic systems are being widely investigated [174]. Attention is also focused on drugs involved in abolishing inflammation in the CNS [178]. The discovery of the antidepressant effect of ketamine has led to the development of research into its use in this therapeutic direction [1].

## 9. The Mechanism of Antidepressant Activity of Ketamine

**Figure 2:**
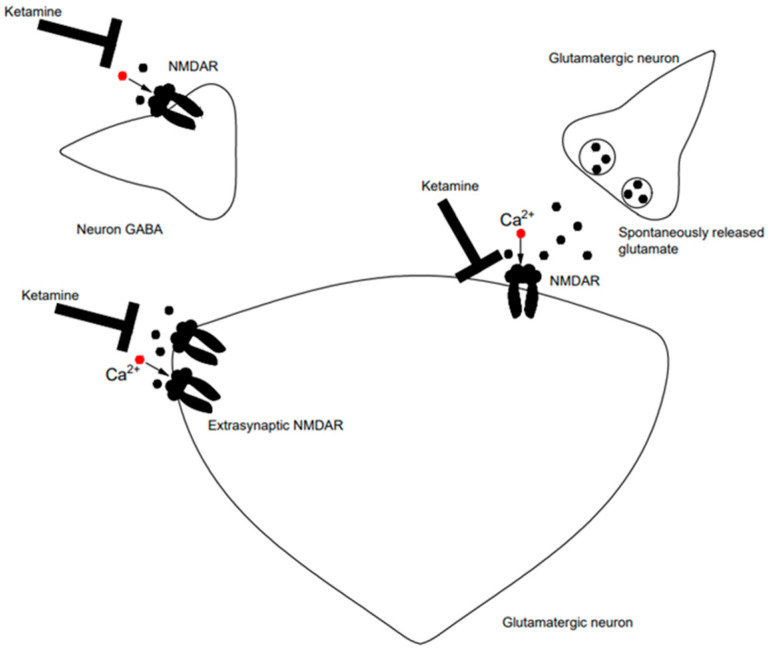
Mechanism of action of ketamine, adaptation based on [19].

In the context of antidepressant action, ketamine selectively blocks NMDA receptors on GABA-ergic interneurons, which results in the stimulation of various neurotransmission in other regions involved in mood regulation (Figure 2). The decrease in GABA release reduces the inhibitory effect of GABA on the glutamatergic system and causes increased presynaptic glutamate secretion. Glutamate then stimulates AMPA receptors [7] and this effect may be responsible for the antidepressant activity of ketamine (but not other antidepressants, suggesting a unique mechanism of action) since the administration of an AMPA receptor antagonist inhibits this effect in behavioral tests. The inhibition of NMDA receptor-dependent spontaneous impulsation in the hippocampus may also be associated with antidepressant effects. This action leads to changes in protein synthesis and increased neurotransmission in the CA1 region of the hippocampus. Ketamine can inhibit extrasynaptic NMDA receptors on pyramidal neurons and thus prevent their activation by decreasing glutamate levels in the extracellular space. This leads to disinhibition of the mechanistic target for rapamycin (mTOR) protein synthesis, which ultimately induces an antidepressant effect. Furthermore, NMDA receptors on pyramidal neurons mainly contain GluN2B subunits, and in mice lacking GluN2B, no antidepressant effect was observed. Additionally, in humans, the administration of selective inhibitors of the GluN2B subunit of NMDA receptors induces antidepressant effects [8]. The antagonism of NMDA receptors leads to the inhibition of lateral habenula (LHb) neurons’ activity [8]. This region is involved in the formation of reward, aversion emotion and cognitive processes [179] and may be activated under aversive environmental stressors, which significantly promote the manifestation of depressive symptoms. The inhibition of LHb activity by ketamine has been found in vitro, while in vivo studies in rats have shown an acute antidepressant effect correlated with these properties [8].

However, there are also studies demonstrating mechanisms independent of NMDA receptor inhibition [7], as other NMDA receptor antagonists do not show antidepressant effects as rapid and intense as those of ketamine [8,180]. In addition, partial agonists such as rapistenol and D-cycloserine are devoid of ketamine’s side effects associated with NMDA receptor inhibition. Furthermore, R-ketamine has an approximately four-fold lower affinity for NMDA receptors than S-ketamine, but induces more potent and longer-lasting antidepressant effects. Researchers also highlight the role of its metabolite S-HNK, while chemically modified ketamine, with reduced metabolism to S-HNK and retaining affinity for the NMDA receptor, induced no antidepressant effects in behavioral tests. Additionally, in this study, a stronger antidepressant effect was observed in rat females with higher brain exposure to S-HNK, while there were no differences in ketamine concentrations between the two genders [8,180].

Other neurochemical pathways involving elongation factor kinase 2 in eukaryotic cells (eEF2K) and BDNF levels are also thought to be involved in this mechanism of action [8]. Ketamine can increase BDNF levels by affecting eEF2K activity. It has been demonstrated that BDNF is essential for the antidepressant activity of ketamine, and mutations in genes crucial for the secretion and activity of this factor in humans and mice resulted in a lack of antidepressant effect. Furthermore, it determines neuroplasticity and influences the formation of synaptic connections. Active eEF2K, regulated by NMDA receptor activity, inhibits the substrate elongation factor in eukaryotic cells (eEF2) which inhibit the translation of BDNF. Ketamine, by inhibiting spontaneous NMDA receptor neurotransmission, deactivates eEF2K, while subsequently activating eEF2 and suppressing protein synthesis. Previous behavioral tests showed that the administration of eEF2K inhibitors to mice reduced depressive symptoms. In addition, no increase in drug-induced BDNF levels was observed in animals lacking genes involved in eEF2K expression. However, attention is drawn to the finding that the effect of ketamine in this case may also be NMDA receptor-independent. The peripheral administration of the ketamine metabolite HNK also causes eEF2 activation and an associated increase in BDNF levels in NMDA receptor-independent mechanisms. Protein synthesis can also be affected by mTOR kinase which is involved in the regulation of neurogenesis, initiates protein translation and synthesis and is crucial for the regulation of synaptic plasticity. Its action is dictated by activation of the mTOR1 complex [8]. The induction of pathways involving mTOR can occur as a result of an increase in BDNF levels, the activation of the receptor for this factor or the activation of tropomyosin kinase (TrkB) receptors by BDNF [8,180]. Interestingly, it was observed that the administration of a selective mTOR inhibitor abolishes the antidepressant effect of ketamine and the synaptic changes. It is thought that activation of the mTOR pathway may be connected to the inhibition of another factor—glycogen synthesis kinase-3 (GSK-3). Mutation in GSK-3-relevant genes prevents activation of the mTOR pathway, but, interestingly, this is not observed after the administration of GSK-3 inhibitors. This situation may be explained by attributing ketamine’s function to the activation of AKT kinase (protein kinase B), which, in part, regulates GSK-3 activity, since it was observed that the inhibition of AKT activity abolishes the antidepressant effect of ketamine (inhibiting GSK-3 and mTOR activity) [8].

## 10. Current Position of Ketamine in the Treatment of Depression

**Figure 3:**
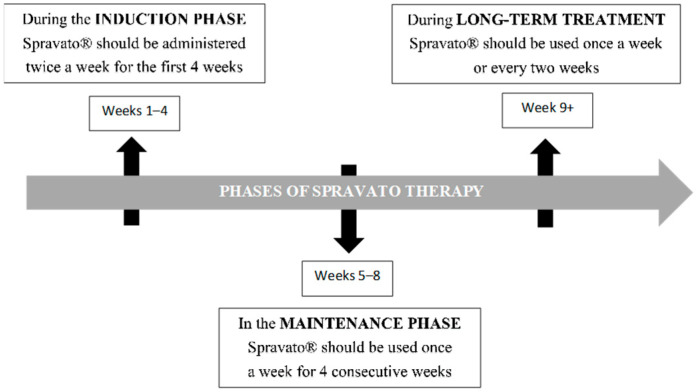
Method of Spravato^®^ administration [184].

Both the racemic form and its two independent enantiomers produce surprising improvements in patient mood. As a result, Johnson & Johnson successfully formulated S-ketamine in an intranasal form (Spravato^®^), which was subsequently approved by the FDA in 2019 for the treatment of TRD in the USA and Europe [179]. In 2020, it was approved also for the treatment of MDD with associated suicidal thoughts or behavior [181]. Furthermore, the i.v. administration of racemic ketamine is used off-label for the treatment of depression [182]. Therefore, S-ketamine is not a drug of first or second choice, but is recommended in situations where time is of the essence, e.g., suicide risk or hospitalization. Nonetheless, its great advantage is its fast antidepressant effects, observed after a few hours or days. In contrast, the lack of sufficient long-term data on its safety and effectiveness raises concerns. Spravato^®^ is only used in adult patients and they self-administer it under direct supervision to reduce the symptoms of depression, together with another antidepressant. It is prescribed when the patient has tried at least two other antidepressants without improvement (Figure 3). A doctor or other healthcare professional will monitor the patient each time they use Spravato^®^. The preparation contains 28 mg of S-ketamine per device (dispenser). During each session, the patient receives from one to three dispensers. The dose is determined individually, most often it is 56 mg, i.e., two dispensers. Administration takes a few minutes (the medicine is administered every 5 min). The patient remains in the facility under observation, for at least 2 h, until any side effects such as disturbances of consciousness and blood pressure, dizziness and headaches, nausea, vomiting, or anxiety disappear. It is not recommended for patients to drive or operate any machines until the end of the day after the administration of the drug. The entire treatment is supervised by a psychiatrist [183].

## 11. The Antidepressant Effect of Ketamine in Animal Studies

In animal studies, the antidepressant effect of ketamine has been shown in a variety of behavioral tests [114]. In a group of rats receiving ketamine or desipramine intraperitoneally (i.p.), Koike and colleagues observed a decrease in the number of failures in the learned helplessness test and a reduction in the time of immobility in the tail suspended test (TST) 30 min after administration. However, only the administration of ketamine obtained similar results in tests performed 72 h after administration, indicating its long duration of action compared to desipramine. Furthermore, ketamine did not affect the locomotor activity of the animals [185]. Subsequently, Jiang et al. demonstrated the antidepressant effect of ketamine in rats 24 h after administration in the standard penetration test (SPT), FST, elevated plus maze test (EPM) and water maze test (WMT), but the effect did not persist with long-term treatment after 7 weeks [186]. However, it should be emphasized that in a group of growing rats, the drug showed stronger and longer-lasting effects as determined by increased locomotion compared to adult rodents, which may suggest a higher potential for addiction in juveniles [187]. In mice, an antidepressant effect was observed in the forced swim test (FST) [64]. In addition, ketamine administration increased saccharose preference in test animals, indicating a reduction in the anhedonia often associated with depression. Moreover, a comparative study of the two enantiomers showed a stronger antidepressant effect of R-ketamine than S-ketamine. In addition, for S-ketamine, a single administration caused a decrease in PV-positive cells in the brain which is associated with psychotomimetic effects and impaired cognitive function, but the changes were not observed for R-ketamine. This evidence may indicate a potential role for R-ketamine in the treatment of depression since it does not induce psychotomimetic effects [63]. Interestingly, in animal models, some researchers have demonstrated neurotoxic effects of ketamine, but no such effects have been found in humans. Furthermore, many of the results from animal studies are inconclusive and have not been reflected in subsequent clinical studies, and thus should be interpreted with caution [188].

## 12. Ketamine in Clinical Trials

Analysis of the clinical trials conducted to date demonstrates the efficacy of ketamine in the treatment of TRD [189]. The antidepressant effect in most patients begins within 24 h of administration and persists for approximately 1 week [190]. Satisfactory effects of pharmacotherapy have been found in both groups: low- and high-resistance patients treated with standard antidepressants. For intravenous ketamine, the effective antidepressant effect in people with lower levels of resistance (resistance up to three antidepressants) is achieved at a dose of 0.5 mg/kg. However, in a group of resistance above three drugs, it is suggested that the dose needs to be increased to 0.75 mg/kg to achieve a long-lasting effect. Studies conducted in different age groups showed an increase in efficacy in patients with an earlier age of onset (<55 years). However, patients in the 65–74 age range also showed satisfactory improvement. On the contrary, a decrease in the effect of ketamine was observed in the age group > 75. These studies indicate a better prognosis when ketamine treatment is initiated earlier when resistance to standard treatment regimens is suspected [189]. Ketamine has also been shown to effectively abolish suicidal thoughts often associated with depression and incidents of hospitalization. To reduce anxiety, standard treatments include benzodiazepines such as midazolam, which some patients do not respond to. Patients unresponsive to midazolam have been shown to experience rapid improvement after i.v. ketamine. The researchers concluded that the use of ketamine would significantly reduce the time and number of hospitalizations and associated costs [191]. However, in the study of Zhang and Hashimoto, 2022 [181], patients showed a similar response to treatment after i.v., s.c. and i.m. administration. After oral administration, the antidepressant effects were slower and observed only after 14 days of use, which may be related to the lower bioavailability, while a slightly higher bioavailability is reported with sublingual administration [190]. Notwithstanding, this form of administration requires further study, but the results to date indicate its efficacy and safety [192].

Additionally, clinical trials using S-ketamine as a treatment were run for patients enrolled in the Risk Evaluation and Mitigation Strategy (REMS) program [193], and the efficacy of each of the doses currently in use was confirmed in phase II clinical trials. These were multicenter, double-blind, placebo-controlled studies. Evaluation was based on the difference in pre- and post-treatment scores on the Montgomery–Asberg Depression Rating Scale (MADRS) [193]. Phase III studies evaluated the efficacy and safety of S-ketamine spray together with a newly included oral antidepressant [193]. The initiative was supported by the FDA based on patient experience and preference data and an analysis of possible harms and benefits. The data came from patients entered in the phase III trial who had already had experience with ketamine and from patients who had not taken it, and was obtained through an online patient portal [189]. Subsequently, the short-term TRANSFORM studies were aimed at assessing efficacy and safety. Depression severity was assessed using the MADRS scale. The TRANSFORM-1 and TRANSFORM-2 study additionally considered the patients’ level of irritability, which affects 30–50% of all patients struggling with a major depressive episode and exacerbates the escalation of suicidal thoughts. The level of irritability was assessed using the 7-item Generalized Anxiety Disorder Scale (GAD-7). The newly included oral antidepressants were duloxetine, escitalopram, sertraline and extended-release venlafaxine [177]. The TRANSFORM-3 study was conducted following the same regimen as TRANSFORM-2 with flexible dosing and included only elderly subjects aged ≥65 [193,194,195,196,197] (Table 5 and Table 6).

A greater improvement was observed in the group receiving S-ketamine plus another antidepressant than in the placebo group. It was more pronounced in those aged 18–64 than in those > 64 years. Additionally, S-ketamine was more effective in those with an earlier age of onset (<55) [196,197]. Likewise, extended clinical trials have assessed the efficacy and safety of long-term S-ketamine use, including the potential for addiction and withdrawal symptoms [193,198].

SUSTAIN-1 focused on assessing long-term efficacy [193]. Basic information about the study conducted is assembled in Table 7. The recurrence of relapse after S-ketamine treatment is shown in Table 8.

The researchers estimated that treatment with S-ketamine reduces the risk of relapse by 51% in those with stable remission and by 70% in those with a stable response, compared to placebo [190].

SUSTAIN-2 was conducted on adults of all ages using flexible S-ketamine dosing [198]. Basic information about the study conducted is assembled in Table 9. A summary of the important results and adverse effects is provided in Table 9, Table 10 and Table 11.

In this study, patients were also administered a second antidepressant. The trial included an induction phase (4 weeks) in which S-ketamine was administered twice a week, a maintenance phase (48 weeks) in which patients received this drug once a week and then a follow-up phase of up to one year in which patients did not receive S-ketamine and continued treatment with another antidepressant [124]. S-ketamine proved to be effective in treating depression, but, importantly, some patients only achieved remission during the maintenance phase [124]. Similarly, in the ASPIRE and ASPIRE II long-term study, not all patients showed an early response to treatment. The greatest response was found on day 25 of the study (74.6%). After 24 h, only 35.3% of all patients showed a response. In the retrospective, multicenter observational study REAL-ESK without a placebo, 38% of all patients not showing a response after one month, had a remission after three months of treatment [126]. This shows that S-ketamine, which initially appears to be ineffective in some people, may produce satisfactory results after prolonged use, and that a lack of response after the initial doses is not an indication for discontinuing pharmacotherapy [127].

In all short-term studies, adverse effects were more frequent in groups receiving S-ketamine plus an antidepressant than in the placebo group, and these effects were classified as mild or moderate. There were no significant differences between the 56 mg S-ketamine dose group and the 84 mg dose group [120]. The most common symptoms in patients aged 18–64 are shown in Table 11.

In the TRANSFORM-3 study, adverse effects were reported by 70.8% of all patients receiving S-ketamine and 60% of all patients in the placebo group. The most common reports were dizziness (20.8%), nausea (18.1%) and dissociation (12.5%). An increase in blood pressure occurred in 12.5% of all patients in the S-ketamine plus antidepressant group and resolved over a 2 h period in most patients. There were no changes in laboratory parameters, electrocardiogram or nasal mucosa. Moreover, no cases of bladder interstitial inflammation occurred [197]. The adverse effects in the long-term study were similar to those observed in the short-term study and had the same severity (Table 12 and Table 13) [198,199,200,201].

It should be emphasized that none of the patients asked for an increase in the dose of the drug and there was no recorded desire to obtain the drug on their own (Table 10) [127]. Two patients died during the study; however, this was found to be unrelated to the treatment [201].

The prospective STRIVE evaluation examined overall patient satisfaction with S-ketamine treatment in terms of mood and effects on functioning. It included patients who had previously participated in clinical trials such as TRANSFORM and SUSTAIN. Patients gave positive feedback on S-ketamine treatment and judged it significantly better compared to previous conventional pharmacotherapy [202]. One study [203] extended the spectrum of research to use ketamine in the prevention of postpartum depression. The results show a decrease in postpartum depression in women who received a low dose of ketamine i.v. (0.5 mg/kg + 2 mL physiological salt) after the postpartum period. The 138 women were divided into two equal groups. The control group received physiological salt only. Women who received ketamine had lower Edinburgh Postnatal Depression Scale (EPDS) scores and significantly fewer incidences of postnatal depression. Ketamine also appeared to be safe and there were no significant differences in side effects between the two groups [132].

Undoubtedly, S-ketamine in the form of a nasal spray is of great interest and so far is the only form registered for the treatment of depression [182]. It can be used by patients with the assistance of medical staff and, in the future, offers the possibility for use in outpatient therapy [199]. Despite the proven efficacy of S-ketamine in clinical trials, treatment-related side effects are still an issue. Recently, R-ketamine has become of interest to researchers [105]. Although it has a lower affinity for NMDA receptors, in rodent studies, it showed a stronger and longer-lasting antidepressant effect than racemic ketamine and S-ketamine. Importantly, this fact did not correlate with an increase in the potency of adverse effects. R-ketamine showed a lower psychotomimetic and dissociative potential than S-ketamine [181,204], presumably as a result of its lower ability to stimulate dopamine release [30]. Based on the available results, it is believed that the antidepressant effect of R-ketamine is not related to NMDA antagonism [181,204], since R-ketamine induces activity within different brain structures than S-ketamine, (R,S)-ketamine and an NMDA antagonist in magnetic resonance imaging (fMR) [205]. However, in the first open pilot study in patients with MDD, R-ketamine showed higher response and remission rates compared to (R,S)-ketamine and S-ketamine. Furthermore, it induced only minor side effects observed as a transient visual impairment and dizziness. Notwithstanding, due to the small size of the study group and the lack of a reference sample, further studies on its clinical efficacy and safety are needed, but, nevertheless, the results obtained seem promising [204].

## 13. Summary

Ketamine, originally used for anesthesia, has gained significant attention in recent years for its potential in treating pain, depression and addiction. In the context of pain management, ketamine may be an alternative treatment for patients with opioid-resistant pain. Additionally, ketamine produces rapid and robust antidepressant effects, especially in patients with TRD. Current research has indicated that LDK can provide fast-acting relief, often within hours, through the modulation of NMDA receptors and increased synaptic plasticity. This has positioned ketamine as a promising therapeutic option for patients who do not respond to conventional antidepressants. In the realm of addiction, ketamine’s ability to disrupt maladaptive neural circuits offers hope for treatment strategies in substance use disorders. Studies suggest that ketamine-assisted psychotherapy supports a reduction in craving and promotes abstinence, making it a potential adjunct in addiction treatment; however, further clinical trials are needed to confirm this efficacy.

Despite these promising findings, further studies are essential to fully understand the long-term effects of ketamine administration, including its safety profile and potential for abuse. Investigating its mechanisms at a molecular level (including the role of its metabolites), the sustainability of its therapeutic effects over time and its neuroprotective effects could open new treatment possibilities for patients with limited therapeutic options.

## Figures and Tables

**Table 1:** Basic pharmacokinetic parameters of ketamine enantiomers (S-, R- and racemic) [34,35,36,37,38].

Ketamine Enantiomer	Route of Administration	Dose (mg/kg)	Bioavailability (%)	Literature
(S-, R-)- ketamine	i.v.	1–4.5	100	[34]
	i.m.	8–10	93	[32,33,35]
	p.o.	0.25–0.5	17–29	[36,37,38,39]
	intranasal	0.3–9.0	45–50	[8,40,41,42,43,44]
S-ketamine	i.v.	0.125–0.3	100	[40,45,46,47,48]
	i.m.	0.27	100	
	p.o.	0.25–0.3	8–24	
	intranasal	0.33	70	
R-ketamine	i.v.		lack of data	[49,50,51]
	i.m.	0.5	93	
	p.o		lack of data	
	intranasal		lack of data	

**Table 2:** Use, contraindications and notes for ketamine [32,66,73,74,75].

Indications	Contraindications	Additional Notes
Anesthesia: Sedation (mechanical ventilation) Procedural sedation Induction and maintenance of general anesthesia Anesthesia for patients with respiratory conditions Anesthesia where hemodynamic balance is required Subanesthesia	Psychosis Poorly controlled hypertension Severe liver dysfunction Epilepsy Cranial injuries (not fully studied) Schizophrenia	Required during administration and procedures: blood pressure, electrocardiogram and respiratory measurements
Analgesia: Acute pain Chronic pain Neuropathic pain Perioperative pain Cancer pain	Severe liver dysfunction Active substance abuse Ulcerative cystitis Significant coronary disease Psychosis	Simultaneous administration of benzodiazepines or α2-adrenoceptor agonists may be required Precaution against chronic postoperative pain—not effective

**Table 4:** Basic characteristics about the included studies.

Author	Schedule of Ketamine Administration	Principal Conclusions
**Ethanol addiction**		
Dakwar, E. et al., 2020 [158]	i.v. administration 0.71 mg/kg + motivational enhancement therapy/psychotherapy	increased likelihood of abstinence, delayed time to relapse and reduced likelihood of heavy drinking days compared to midazolam
Das et al., 2019 [159]	i.v. 0.5 mg/kg for 10 days	reduction in the reinforcing effects of ethanol, reduction in number of drinking days per week and volume of consumed alcohol
Grabski et al., 2022 [160]	i.v. 0.8 mg/kg + psychotherapy	increase the number of abstinent days
Kolp et al., 2006 [148]	i.m. administration 2–3 mg/kg + psychotherapy	extending the period of alcohol abstinence
Krupitsky et al., 1992 [161]	i.m. 3 mg/kg + psychotherapy	extending the period of alcohol abstinence
Krupitsky and Grinenko, 1997 [149]	i.m. 2.5 mg/kg + psychotherapy	extending the period of alcohol abstinence and reduced risk of relapse
Pizon et al., 2018 [162]	i.v. 0.15–0.3 mg/kg/h + bolus (0.3 mg/kg) + conventional withdrawal treatment	reduction in benzodiazepine requirements, decrease likelihood of intubation and a shorter length of stay in the intensive care unit (ICU)
Rothberg et al., 2021 [163]	i.v. 0.71 mg/kg + motivational enhancement therapy	increased probability of abstinence, delayed time to relapse, decreased likelihood of heavy drinking days compared to midazolam
Shah et al., 2018 [164]	i.v. 0.75 mg/kg + conventional withdrawal treatment	enhanced symptom control for benzodiazepine-refractory patients and reduced infusion requirements
Yoon et al., 2019 [165]	i.v. 0.5 mg/kg once a week for 4 weeks + naltrexone 380 mg	reduced alcohol craving and consumption
Wong et al., 2015 [166]	i.v. median infusion 0.20 mg/kg/h + conventional withdrawal treatment with a standardized treatment protocol (benzodiazepine + dexmedetomidine + phenobarbital + propofol ± antipsychotics + clonidine + intubation)	reduction in short-term benzodiazepine dose requirements in patients with alcohol withdrawal
**Cocaine addiction**		
Dakwar et al., 2014 [150]	3 × i.v. 0.41 mg/kg or 0.71 mg/kg	enhanced motivation to quit and dampened cue-induced craving
Dakwar et al., 2017 [167]	i.v. 0.11 mg/kg 2-min bolus + 0.60 mg/kg 50 min	decreased cocaine self-administration
Dakwar et al., 2018 [168]	i.v. 0.71 mg/kg	decreased cocaine self-administration, cocaine use and craving
Dakwar et al., 2019 [158]	i.v. 0.5 mg/kg	promoted abstinence, diminished craving and reduced risk of relapse
**Opioid addiction**		
Jovaiša et al., 2006 [169]	i.v. 0.5 mg/kg	better control of withdrawal symptoms with no effects on treatment of opiate dependence after 4 months
Krupitsky et al., 2002 [153]	i.m. 0.2 or 2.0 mg/kg + psychotherapy	increased rate of abstinence within the first two years of follow-up, reduction in craving for heroin, positive change in nonverbal unconscious emotional attitudes
Lalanne et al., 2016 [170]	oral administration, 1 mg/kg	reduction in dosage of opioid painkillers without withdrawal symptoms
Omoigui et al., 2011 [171]	i.v. 5 mg/kg	effective treatment for the opioid withdrawal symptoms and pain during transition to buprenorphine
Pradhan and Rossi, 2020 [172]	i.v. 0.75 mg/kg	Combination therapy with ketamine, rTMS and TIMBER is feasible in patients with opioid addiction, reduces craving and promotes abstinence

**Table 5:** Essential information about the TRANSFORM clinical trial.

	TRANSFORM-1	TRANSFORM-2	TRANSFORM-3
Features of a clinical trial	Randomized, double-blinded and placebo controlled		
Number of respondents included in the analysis	324	223	137
Age of respondents	18–64	18–64	≥65
Basic selection criterion	Recurrent MDD or an episode of MDD lasting ≥ 2 years without psychotic features	Recurrent MDD or an episode of MDD lasting ≥ 2 years without psychotic features	MDD without psychotic features and resistant to ≥2 different AD
Dosage in groups	56 mg or 84 mg of S-ketamine + AD, placebo + AD	56 mg or 84 mg of S-ketamine + AD, placebo + AD	28 mg, 56 mg or 84 mg of S-ketamine + AD, placebo + AD
Duration of treatment phase	28 days	28 days	28 days

**Table 6:** Significant results from clinical trials TRANSFORM.

		TRANSFORM-1	TRANSFORM-2	TRANSFORM-3
**Initial average score on the MADRS**		37.55	37.15	35.2
Mean change in MADRS score	S-ketamine	−18.9	−21.4	−10
	Placebo	−14.8	−17	−6.3
Mean change in irritability on the 7-GAD scale	S-ketamine	−7.4	−7.9	-
	Placebo	−6	−6.8	
Summary response and remission by day 28	S-ketamine	53.6%	69.3%	44.4%
	Placebo	38.9%	52%	20%

**Table 7:** Essential information about the SUSTAIN-1 clinical trial.

SUSTAIN-1	
**Main objective of the clinical trial**	Long-term effectiveness
Features of the clinical trial	Randomized, double-blinded, placebo controlled
Number of respondents	705
Age of respondents	18–64
Basic selection criterion	Recurrent MDD or an episode of MDD lasting ≥ 2 years without psychotic features. No suicidal thoughts or behavior.
Duration analyzed	Induction phase (4 weeks) Optimization phase (12 weeks) Sustaining phase (variable duration)
Dosage	Variable dosage 56 mg or 84 mg S-ketamine with an AD. Induction phase—twice a week Optimization phase—one or two times a week Sustaining phase—AD only

**Table 8:** Relapse in the group with stable remission and stable response.

Relapse	S-ketamine + AD	Placebo + AD
Patients with stable remission [%]	26.7	45.3
Mean time to relapse in stable remission in days	635	88
Patients with stable response [%]	25.8	57.6

**Table 9:** Essential information about the SUSTAIN-2 clinical trial.

SUSTAIN-2	
**Main objective of the clinical trial**	Long-term safety and effectiveness
Features of the clinical trial	Non-randomized, no placebo
Number of respondents	802
Age of respondents	≥18
Basic selection criterion	MDD without psychotic features and resistant to ≥2 different antidepressants. No suicidal thoughts or behavior.
Duration analyzed	Induction phase (4 weeks) Sustaining phase (48 weeks) Observation phase (up to one year)
Dosage	Flexible dosage of 28 mg (in age of ≥65), 56 mg or 84 mg S-ketamine with an AD Induction phase—twice a week Sustaining phase—one or two times a week Observation phase—AD only

**Table 10:** Changes in MADRS and 7-GAD score in the SUSTAIN-2 clinical trial.

		Result
**Mean baseline MADRS score**		31.4 ± 5.39
Mean change in the MADRS score		−16.4
Clinical response of respondents (↓MADRS ≥ 50%)	Induction phase	78.4%
	Optimization/observation phase	76.5%
Remission of respondents (MADRS ≤ 12)	Induction phase	47.2%
	Optimization/observation phase	58.2%
Mean change in 7-GAD score	Induction phase	−5.9
	Optimization/observation phase	0.2

**Table 11:** Summary of mean incidence of significant adverse event in TRANSFORM-1 and TRANSFORM-2 clinical trials. AD—antidepressant.

	Total S-Ketamine + AD (343 Participants)	Total Placebo + AD (222 Participants)
	Incidence of Adverse Event [%]	
**Adverse event**	27.8	8.5
Nausea	26.5	3.6
Dissociation	22.6	6.7
Dizziness	20.2	17.1
Headache	16.3	9
Somnolence	20.2	13.5
Dysgeusia	8.9	2.2
Blood pressure increased	27.8	8.5

**Table 12:** Frequency of TEAE and TEAE suggesting abuse in the SUSTAIN-2 clinical trial.

Adverse Effects	[%]
Prevalence TEAE	90.1
≥1 serious TEAE	14.7
Serious TEAE associated with increased pressure	12.8
TEAE suggesting abuse	53.5
Overdose	Not reported
Abuse of S-ketamine	
Request of increased dosage	
Attempting to get the drug	

**Table 13:** Most common adverse effects in patients in the SUSTAIN-2 clinical trial during S-ketamine use.

	[%]
Dizziness	32.9
Headache	24.9
Cognitive impairment	Not reported
Somnolence	16.7
Dysgeusia	11.8
Dissociation	27.6
Nausea	25.1
Vomiting	10.8
Urinary tract infection	8.1
Bladder inflammation	Not reported
Increase in blood pressure	9.4
